# Supplementary material for: Assessing the Efficacy of Dietary Selenomethionine Supplementation in the Setting of Cardiac Ischemia/Reperfusion Injury
Source: Antioxidants (Basel). 2019 Nov 13;8(11):546. doi: 10.3390/antiox8110546 (PMC6912310; doi:10.3390/antiox8110546)
Supplement: Supplementary file 1 [file antioxidants-08-00546-s001.pdf]

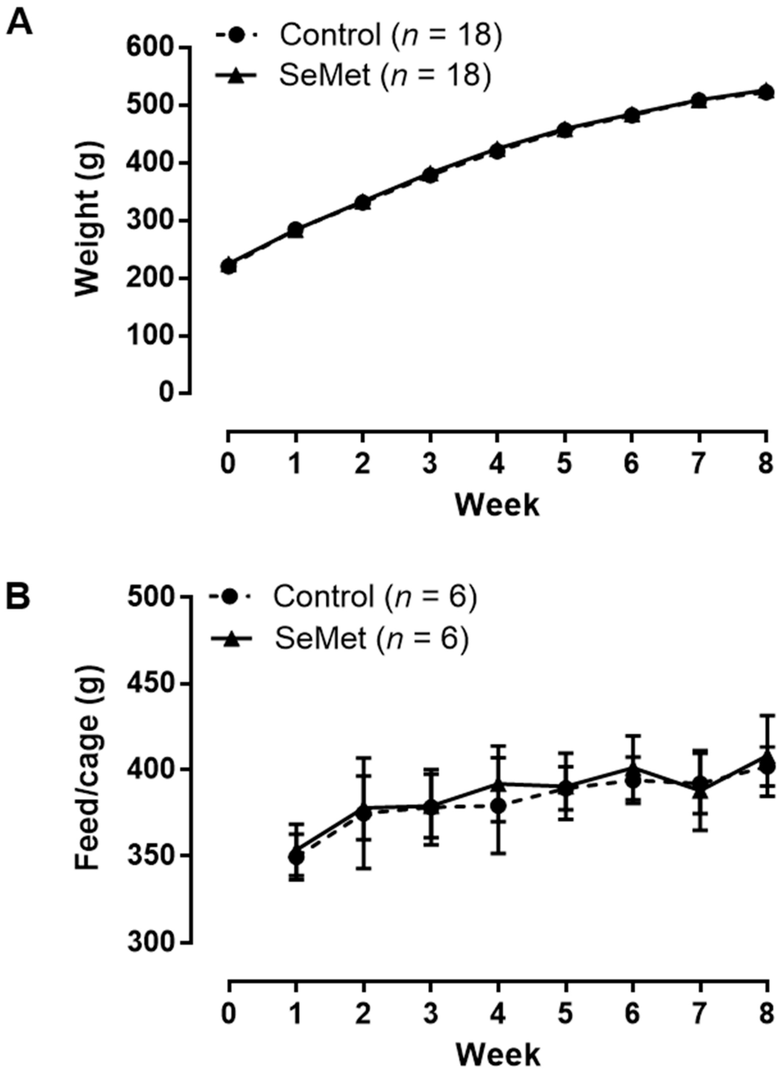

Supplementary Figure 1. Body weight and food intake of rats on a SeMet-supplemented diet. *Male Wistar rats (100-125 g) were housed two rats per cage and randomly assigned into groups receiving either normal chow (control, dashed line) or normal chow supplemented with SeMet (2 mg kg*$^{-1}$*, solid line) ad libitum for 8 weeks, with the (A) body weight of rats and (B) food consumption recorded at the end of each week. No significant difference between control and SeMet-supplemented group as determined by two-way ANOVA with Holm-Sidak post-hoc testing.*
